# The Emerging Role of Citrulline and Theanine in Health and Disease: A Comprehensive Review

**DOI:** 10.3390/nu17213496

**Published:** 2025-11-06

**Authors:** Xiaokang Lv, Chao Chen, Yan Liang, Yating Song, Jie Liu, Wenxun Chen, Hao Li

**Affiliations:** 1 College of Animal Science, Anhui Science and Technology University, Chuzhou 233100, China; lvxk@ahstu.edu.cn (X.L.); chenchao@ahstu.edu.cn (C.C.); liangy@ahstu.edu.cn (Y.L.); 13696653684@163.com (Y.S.); 19565982716@163.com (J.L.); 2 Hubei Key Laboratory of Animal Nutrition and Feed Science, Wuhan Polytechnic University, Wuhan 430023, China; 3 School of Biotechnology, Jiangsu University of Science and Technology, Zhenjiang 212100, China

**Keywords:** L-citrulline, L-theanine, immunity

## Abstract

Non-proteinogenic amino acids, such as L-citrulline and L-theanine, have garnered attention for their potential health benefits, including enhanced immunity, antioxidant activity, and cardiovascular support. The application of natural amino acids in disease treatment and health supplementation is and will remain a research hotspot in pharmaceutics. Plant-derived L-citrulline and L-theanine have demonstrated multifaceted benefits, primarily through mechanisms involving nitric oxide (NO) bioavailability (for L-citrulline) and mitochondrial regulation or immune modulation (for both). Critical gaps are identified: (1) the role of D-amino acids (e.g., D-citrulline and D-theanine) in health and metabolism remains underexplored, particularly regarding chiral-specific bioactivity; (2) derivatives and co-administration strategies of L-forms warrant systematic evaluation for drug. However, while these compounds show promise, evidence is predominantly from animal and cell studies, with limited long-term human data on efficacy and safety. Potential side effects, dosing limitations, and sourcing challenges are discussed. This review emphasizes the need for cautious interpretation of their benefits, acknowledging that while promising, some effects, such as those on muscle protein synthesis, require further validation compared to established nutrients like branched-chain amino acids. By bridging mechanistic insights with translational challenges, this work aims to guide future research toward sustainable nutraceutical production.

## 1. Introduction

Amino acids are the building blocks of proteins. They play an extremely important role in the activities of living organisms, especially humans. Amino acids are responsible for the operation of the entire human body and ensure the normal functioning of various systems. The human body utilizes 20 standard amino acids, of which 9 are essential (must be obtained from the diet) and 11 are non-essential (can be synthesized endogenously) [1]. Given that dietary amino acid profiles are rarely perfectly balanced, metabolic disorders may arise from prolonged imbalances, which have been associated with various pathologies including sarcopenia [2], immune dysregulation [3], cardiovascular diseases [4], and cancer [5,6]. For example, branched-chain amino acids (BCAAs) support muscle growth and energy during exercise, while arginine aids nitric oxide production for vascular health [7]. This rationale positions amino acids as promising candidates for addressing metabolic dysregulation. Recent advancements in pharmaceutical sciences highlight amino acids’ dual role as both therapeutic agents and drug delivery enhancers [8,9].

Additionally, selenocysteine, an uncommon amino acid, is incorporated into specific proteins, while over 300 non-proteinogenic amino acids (NPAA), such as L-citrulline and L-theanine, exhibit biological activity without being part of protein structures [10]. Among non-proteinogenic amino acids, L-citrulline and L-theanine were selected for this review due to their unique metabolic pathways, dietary availability, and growing research interest. L-citrulline, abundant in watermelon, is a key intermediate in the urea cycle and enhances nitric oxide (NO) bioavailability, impacting cardiovascular and immune health [11]. L-theanine, found predominantly in tea, modulates immune responses and exhibits antioxidant properties, making it a candidate for neurological and intestinal health applications [12]. Their natural dietary sources and promising preclinical data distinguish them from other non-proteinogenic amino acids, such as ornithine or homocysteine, which have less extensive research or narrower applications [13]. Both natural and synthetic compounds can pose risks, and the safety of amino acids depends on dose, context, and individual factors. For example, natural compounds like amatoxins from mushrooms can be highly toxic, while synthetic amino acid derivatives are rigorously tested for safety [14]. To provide a clear rationale, this review integrates data on both compounds under a unified framework of NPAAs’ roles in health, emphasizing shared themes like immune and metabolic modulation while contrasting their mechanisms. This approach avoids disjointed presentation by using comparative sections and transitions, supported by recent multidisciplinary studies grouping similar NPAAs for stress alleviation and nutraceutical potential [15]. This review systematically examines the mechanistic basis and translational potential of L-citrulline and L-theanine, addressing their benefits and limitations in human health applications.

## 2. Discovery of L-Citrulline and L-Theanine

### 2.1. Natural Occurrence of L-Citrulline

The discovery of L-citrulline dates back 110 years ago when it was first identified by Yotaro Koga and Ryo Ohtake [16] in watermelon juice. It was not until 1930 that L-citrulline was confirmed as an amino acid. L-citrulline is scarce in food, with the most abundant natural source being watermelon (*Citrullus lanatus* (Thunb.)). It also exists in cucumbers, pumpkins, melons, bitter gourds, squashes, and gourds [17,18,19]. L-citrulline can be extracted from other fruits of the Cucurbitaceae family as well. L-citrulline is present in both the pulp and rind of watermelons, with a higher concentration in the rind [20]. Normal plasma levels in healthy adults range from 20 to 40 μmol/L, derived primarily from intestinal glutamine metabolism; deficits occur in conditions like short bowel syndrome or sepsis, where endogenous production from glutamine becomes insufficient due to reduced enterocyte function [21]. Currently, the production of L-citrulline mainly relies on extraction from watermelons. The content of L-citrulline in watermelons typically ranges from 0.7 g/kg to 3.6 g/kg of fresh weight [20]. The content of L-citrulline in watermelon rind is reported to be between 0.18 and 2.41 mg/g of fresh sample [19,22], and the specific content varies depending on the variety of watermelon. L-citrulline is a non-essential amino acid and can be endogenously synthesized by the human body [13]. L-citrulline does not directly participate in the protein synthesis process [23] and initially aroused little interest. However, over time, due to its unique metabolism and beneficial effects, research on L-citrulline has proliferated.

### 2.2. Natural Occurrence of L-Theanine

L-Theanine, systematically designated as N-ethyl-γ-L-glutamine, is a unique non protein amino acid in tea that plays an immunomodulatory role in inflammation, nerve damage, intestinal tract, and tumors [12]. Theanine constitutes over 50% of all amino acids in tea. In fresh tea leaves, all the theanine is L-theanine, while processed tea (green tea, oolong tea, and black tea) contains D-theanine [24]. L-theanine content in tea varies by processing method and type, typically ranging from 1 to 2% of dry weight in fresh leaves (up to 6–7 mg/g on average). Minimally processed teas like green and white retain higher levels (average 6.5–6.3 mg/g), while fermented black and oolong teas have lower amounts (5.1–6.1 mg/g) due to oxidation and heat promoting breakdown or conversion to volatiles [25,26]. Preservation is enhanced with lower fermentation and temperatures during processing, ensuring sufficient L-theanine for biological effects in typical consumption (e.g., 20–40 mg per cup of green tea) [27]. Artificially synthesized theanine encompasses both D and L forms. Within tea plants, L-theanine is synthesized from ethylamine and glutamic acid via the theanine synthetase, and the structure of L-theanine is highly similar to that of glutamic acid [28]. In the human body, D-theanine demonstrates relatively low biological activity, whereas L-theanine exhibits exceptionally high biological activity [29]. Hence, this review primarily focuses on L-theanine. L-Theanine is one of the crucial constituents determining tea quality, featuring a caramel-like flavor, capable of mitigating the bitterness of coffee, and serving as a major contributor to the flavor of tea [30]. L-theanine is a safe and non-toxic substance, thereby enabling its utilization as a food additive and facilitating its facile absorption and utilization by the organism [31]. Baseline plasma levels in humans are low (near undetectable without intake), but consuming 4 cups of green tea (~500 mL) can provide ~100 mg, leading to peak plasma levels of ~25 μmol/L; however, much is metabolized during first-pass in the gut/liver [32]. With the continuous advancement of research, it has been discovered that L-theanine possesses multiple physiological and healthcare benefits, thereby commanding significant attention. Research indicates that L-theanine holds a series of efficacies, such as enhancing the body’s immunity, alleviating fatigue, improving human memory capacity, preventing diseases, and relieving nervous tension [33,34,35].

## 3. Metabolism of L-Citrulline and L-Theanine

### 3.1. Metabolism of L-Citrulline

**Figure 1:**
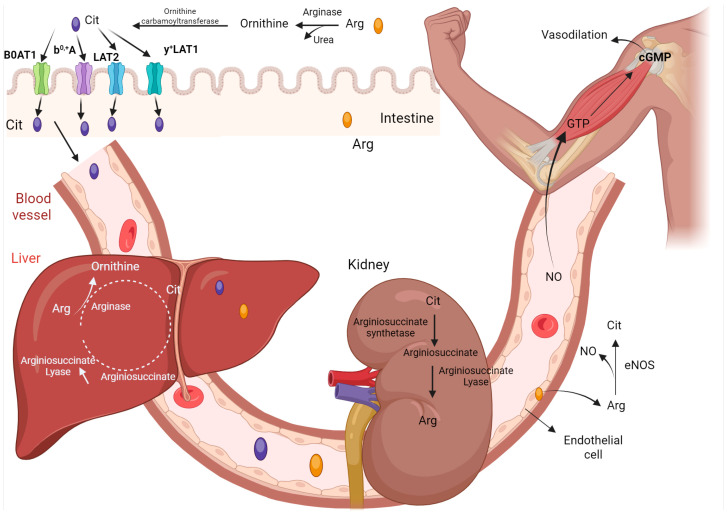
The transport and metabolism of L-citrulline. B0AT1, b0, +A, LAT2 and y+LAT1 are regarded as the potential transport carriers of citrulline in the intestinal tract. Oral administration of L-citrulline is not catabolized by arginase in the intestinal tract, nor can L-citrulline be utilized in the liver. The distinctiveness of L-citrulline resides in its predominant uptake by the kidneys (75%), where it is transformed into ARG and subsequently released. Arg: Arginine; Cit: Citrulline; NO: Nitric oxide.

Concerning the transport mechanism of L-citrulline, it is currently considered that broad neutral amino acid transporter B0AT1 (*solute carrier family 6 member 19*, *SLC6A19*) and b^0,+^A (*solute carrier family 7 member 9*, *SLC7A9*) are regarded as potential transport carriers of L-citrulline in the intestinal tract of the organism [36,37,38] (Figure 1); however, no literature has thus far substantiated this conclusion. Recent research suggests these transporters facilitate uptake based on structural similarities, though direct evidence is limited. Furthermore, based on the transport specificities and structural similarities of proteinogenic AAs (to L-citrulline), large neutral amino acid transporter 2 (LAT2, *solute carrier family 7 member 8*, *SLC7A8*) and y^+^LAT1 (*solute carrier family 7 member 7*, *SLC7A7*) are the prime candidates for the transport of L-citrulline in the intestinal tract [39] (Figure 1). L-citrulline is efficiently absorbed in the small intestine, with high oral bioavailability (~97%) and peak plasma concentrations reached within 0.7–1 h post-ingestion in a dose-dependent manner [40,41]. L-citrulline metabolism is unique. Following the ingestion of arginine (ARG) or glutamine (GLN) by animals, a significant amount of L-citrulline is released in the small intestine. This organ serves as the exclusive source of L-citrulline in systemic circulation [42]. The blood level of L-citrulline may reflect the function and quality of intestinal epithelial cells, and L-citrulline can be utilized as a biomarker for intestinal failure resulting from the reduction in the quality of intestinal epithelial cells [43]. Specifically, plasma levels below 20 μmol/L indicate significant enterocyte mass reduction, with 82% sensitivity and specificity for predicting parenteral nutrition dependence in short bowel syndrome [21]. L-citrulline is the ultimate product of intestinal glutamine metabolism, accounting for 27.6% of the nitrogen of metabolized glutamine [42]. Endogenous production from glutamine is insufficient in states like sepsis or critical illness, leading to deficits [44]. Unlike L-arginine, oral ingestion of L-citrulline is not catabolized by arginase in the intestine, and the enzymatic activity of argininosuccinate synthetase (the enzyme that catabolizes L-citrulline) is relatively low in intestinal epithelial cells [45]. This substrate specificity of arginase for arginine (not citrulline) allows oral L-citrulline to bypass significant intestinal metabolism, entering circulation intact before primary conversion to L-arginine occurs systemically, particularly in the kidneys [41,46]. L-citrulline can inhibit arginase activity and, as an inhibitor, can upregulate the bioavailability of L-arginine in the animal organism [47]. L-citrulline cannot be utilized in the liver either. The uniqueness of L-citrulline lies in its predominant uptake by the kidneys (75%) (Figure 1). Since the kidneys possess argininosuccinate synthetase (ASS) and argininosuccinate lyase (ASL) activities but lack other enzymes of the urea cycle, L-citrulline is transformed into ARG in the kidneys and subsequently released (Figure 1). Given that the majority of ARG is decomposed by the liver (arginase), this inter-organ cycle of ARG-L-citrulline-ARG can safeguard ARG from excessive degradation by the liver. Hence, supplementing L-citrulline constitutes an effective strategy for enhancing the utilization rate of arginine. Muscles do not possess the enzymes (namely, ASS and ASL) for metabolizing L-citrulline into arginine [48] (Figure 1).

Polyamines, such as putrescine, spermidine, and spermine, are critical metabolites derived from arginine via ornithine decarboxylase but not directly from L-citrulline [49]. Polyamines regulate cell proliferation, differentiation, and immune responses, impacting gut health and cancer progression [50]. L-citrulline’s inability to produce polyamines distinguishes its metabolic role from arginine, which serves as a precursor for both NO and polyamines, influencing cellular metabolism differently [51].

### 3.2. Metabolism of L-Theanine

**Figure 2:**
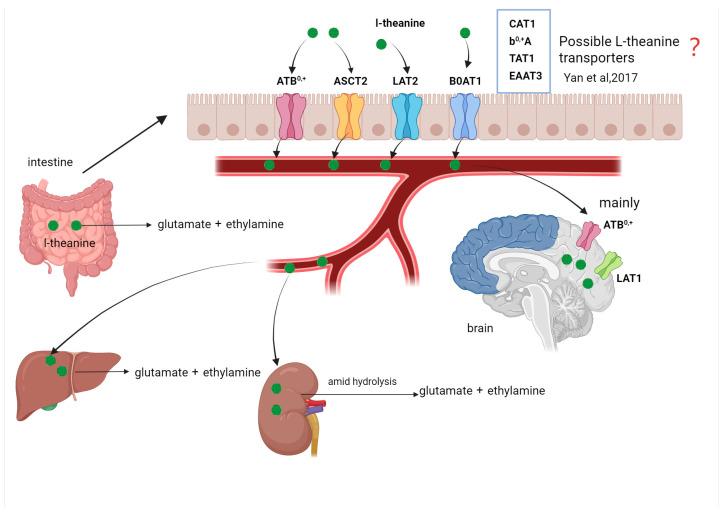
The transport and metabolism of L-Theanine [55]. This figure shows potential intestinal transporters (e.g., B0AT1, ATB0+, ASCT2) for L-theanine uptake, its hydrolysis to ethylamine and glutamic acid in gut/liver/kidney, and brain entry via LAT1/ATB0+.

L-theanine is a water-soluble molecule that can rapidly reach the intestinal tract upon oral administration [52]. L-Theanine is transported from the intestinal brush border into the intestinal epithelial mucosal cells and enters the bloodstream via sodium-coupled active transport proteins. Additionally, it has been discovered that L-Theanine can be transported through the methionine carrier transport system (Figure 2). Vuong et al. [53] asserted that L-Theanine can be transported in the intestine through the methionine transport carrier. Methionine in the intestinal tract is mainly transported via B0AT1 (*solute carrier family 6 member 19*, *SLC6A19*), ATB^0+^ (*solute carrier family 6 member 14*, *SLC6A14*), ASCT2 (*solute carrier family 1 member 5*, *SLC1A5*), LAT1 (*solute carrier family 7 member 5*, *SLC7A5*), LAT2 (*solute carrier family 7 member 8*, *SLC7A8*), etc. [54] (Figure 2). Yan et al. [55] reported that L-Theanine stimulated the expression of CAT1 (*solute carrier family 7 member 1*, *SLC7A1*), b^0,+^A (*solute carrier family 7 member 9*, *SLC7A9*), ASCT2, TAT1 (*solute carrier family 16 member 10*, *SLC16A10*), and EAAT3 (*solute carrier family 1 member 1*, *SLC1A1*) in the intestinal tract of mice, yet it remains unclear whether these transport carriers are implicated in the transport of L-Theanine (Figure 2). While no specific transporter for L-theanine is confirmed, evidence suggests involvement of LAT1 and similar systems based on structural analogy to glutamine; it undergoes partial first-pass metabolism in the gut and liver, where it may be hydrolyzed by glutaminases, with ~50–70% escaping to systemic circulation depending on dose [56]. The transport mechanism of L-Theanine is still rather ambiguous. Research has revealed that L-Theanine can enter the blood–brain barrier through the leucine transport mechanism [52]; however, it is uncertain whether this mechanism is also present in the intestinal tract. The metabolic pathway of L-Theanine within the organism remains unclear. The existing limited studies indicate that L-Theanine in the bloodstream is transported to various organs of the body, predominantly the brain. L-Theanine can be directly excreted through urine or hydrolyzed into ethylamine and glutamic acid in the intestine and liver [57] (Figure 2). Ethylamine from hydrolysis has normal plasma levels of ~1–5 ng/mL; high levels (>50 ng/mL) may be toxic, potentially causing headaches or irritation, though no direct toxicity from theanine-derived ethylamine is reported at typical doses [58]. Hydrolysis can also occur in the kidneys and be converted into glutamic acid and ethylamine by phosphate-independent glutaminase amid hydrolysis, and subsequently excreted from the body along with urine [32] (Figure 2). The transport and metabolic fate of L-Theanine within the organism remain elusive. In the future, efforts should be made to actively explore the transport mechanism and metabolic fate of L-Theanine.

## 4. The Beneficial Effects of L-Citrulline

### 4.1. L-Citrulline—An Excellent Substitute for Arginine in the Treatment of Endothelial Cell Dysfunction

NO participates in the regulation of various biological functions. Endothelial cell dysfunction, which is characterized by impaired endothelium-dependent vasodilation due to the compromised ability of endothelial cells to produce NO, constitutes a major cause of cardiovascular diseases [59]. Furthermore, the early symptoms of type 2 diabetes and hypertension are also dysfunctions caused by the reduced biological availability of NO in endothelial cells [60,61]. L-arginine can generate NO through endothelial nitric oxide synthase (eNOS), and the vast majority of L-arginine utilized for NO production originates from L-citrulline [62]. Consequently, L-arginine and L-citrulline can be employed as supplements to enhance NO production, thereby alleviating endothelial cell dysfunction and treating cardiovascular diseases, diabetes, and hypertension [63]. Human studies support this, e.g., 2–10 g/day reduces blood pressure in hypertensive adults [64]. Within the body, approximately 60% of L-arginine can be synthesized from L-citrulline [65]. When L-arginine is employed as a medication for cardiovascular diseases, it presents the following drawbacks: when the arginine level is excessively high, it will enhance the expression of arginase, thereby resulting in more arginine decomposition, which can increase gastrointestinal irritation, L-citrulline avoids these issues, making it a preferable supplement [66]. Furthermore, in cases accompanied by diseases such as diabetes, trauma, pulmonary arterial hypertension, and heart failure, the expression of arginase increases, significantly reducing the therapeutic efficacy [67]. Hence, L-citrulline can be utilized clinically to substitute L-arginine in the treatment and amelioration of cardiovascular diseases associated with endothelial dysfunction [68]. A recent meta-analysis demonstrates that the intake of L-citrulline is capable of improving cardiovascular function [64].

L-Citrulline as a supplement (10 g/d for a duration of 4 weeks) can reduce aortic pressure in postmenopausal women and diminish the occurrence of cardiovascular diseases [69]. While 10 g/day is a relatively high dose, clinical studies indicate it is safe and well-tolerated for short-term use (up to 4 weeks) in healthy adults, with no significant adverse effects reported at doses up to 15 g/day, though gastrointestinal symptoms may occur at higher intakes due to potential absorption saturation [70,71]. Oral administration of L-citrulline (2000 mg/day for 1 month) can elevate the level of NO in the plasma of patients with type 2 diabetes and decrease arginase activity, thereby conferring benefits to diabetic patients [61]. A recent study discovered that citrulline can ameliorate blood sugar conditions and inflammation in diabetic patients [72]. The ability of citrulline to improve inflammation might be attributed to its capacity to cyclically generate arginine, which possesses anti-inflammatory capabilities [73]. On the other hand, citrulline can reduce inflammatory cytokines and increase anti-inflammatory factors [74]. Furthermore, L-citrulline can be utilized as a pre-exercise supplement to enhance the concentration of arginine in the plasma, thereby promoting the synthesis of NO, augmenting the oxygen and nutrient delivery to muscles, and ultimately enhancing the athletic performance of athletes [13]. In the future, further exploration of the beneficial effects of L-citrulline is requisite, particularly in relation to diseases associated with the biological utilization of NO, such as brain and intestinal health [75]. Currently, our knowledge regarding the role of D-citrulline is extremely limited. Even though the proportion of D-citrulline is lower than that of L-citrulline in the natural state, an early sole study indicated that both D- and L-citrulline were capable of alleviating ischemia/reperfusion-induced myocardial injury in rats [76]. Future research should focus on the physiological metabolism of D-citrulline and its potential influence on immune function.

### 4.2. The Role of L-Citrulline in Regulating Intestinal Immunity

**Figure 3:**
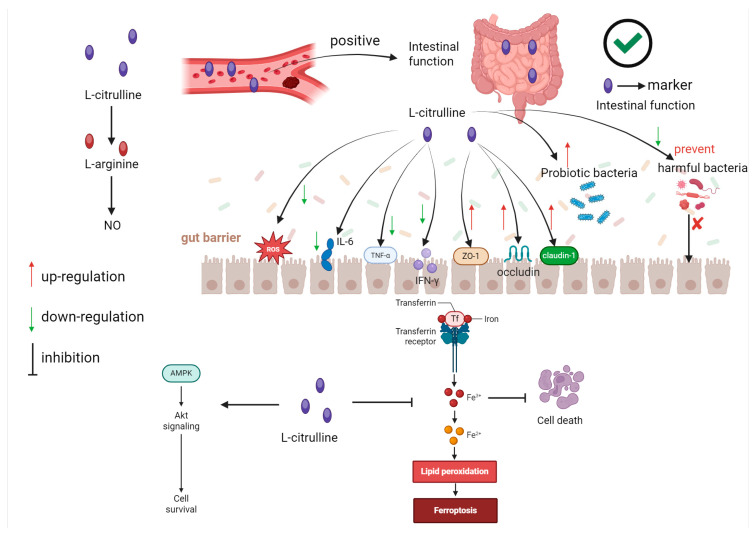
The regulation of intestinal immunity by L-Citrulline. L-citrulline is considered a biomarker for intestinal dysfunction, and the content of L-citrulline in plasma is believed to be positively correlated with the intestinal absorption capacity. The treatment with L-citrulline improves the intestinal barrier and reduces the bacterial translocation in the intestinal mucosa. The improvement of intestinal immunity by L-citrulline is associated with the conversion of L-citrulline to L-arginine, and the supplementation of L-arginine can increase the production of NO. L-citrulline can also decrease the production of pro-inflammatory factors such as IL-6, TNF-α, and IFN-γ, and enhance the expression of tight junction proteins (ZO-1, occludin, and claudin-1). L-citrulline reduces the excessive generation of reactive oxygen species (ROS), inhibits the proliferation of harmful bacteria and promotes the proliferation of beneficial bacteria. L-citrulline suppresses ferroptosis and induces the activation of the AMPK pathway, enhances the intestinal antioxidant capacity, and alleviates intestinal damage caused by iron overload.

The health of the intestine is of paramount importance for the uptake of nutrients by the organism and the overall degree of health. Hence, efforts have been devoted to exploring supplements capable of enhancing intestinal health [77]. Intestinal health includes the integrity of the intestinal barrier and the stability of the microbial community, among others [78]. L-citrulline is considered a biomarker of intestinal dysfunction [79] (Figure 3). The L-citrulline content in plasma is believed to be positively correlated with intestinal absorption capacity and negatively correlated with intestinal injury [80]. L-citrulline treatment can improve the intestinal barrier and reduce the translocation of intestinal mucosal bacteria [81].

Currently, the prevailing opinion suggests that the improvement of intestinal immunity by L-citrulline is associated with its systemic conversion into L-arginine, primarily in the kidneys rather than the intestine, where enzymatic activity for this conversion is low. This leads to increased circulating L-arginine levels that enhance NO production and support intestinal functions [46,82]. The supplementation of L-arginine can enhance the production of NO, which plays a crucial role in modulating immune functions [83] (Figure 3). While promising in animals, human evidence is limited to indirect benefits via NO in conditions like diabetes [61]. Nevertheless, when L-arginine is directly supplemented as a supplement, it might increase the irritation to the gastrointestinal tract [66]. Consequently, L-citrulline might be a more favorable alternative. L-citrulline can diminish the production of pro-inflammatory factors and augment the generation of anti-inflammatory factors. For example, it was discovered that feeding mice induced by dextran sulfate sodium (DSS) with Lactobacillus helveticus-fermented milk rich in L-citrulline could decrease the expression of pro-inflammatory factors such as IL-6, TNF-α, and IFN-γ, and enhance the expression of tight junction proteins (ZO-1, occludin, and claudin-1), thereby alleviating colonic injury [74] (Figure 3). This is preliminary animal data; human trials are needed. The supplementation of L-citrulline increased the production of *IL-10* and *TGFβ* in European sea bass and reduced the expression of pro-inflammatory genes (*IL-1β* and *MMP9*) [84]. Excessive generation of reactive oxygen species (ROS) in the intestine can cause intestinal damage [85]. L-citrulline possesses antioxidant properties [86] and can modify the redox status of the intestinal tract, thereby enhancing intestinal immunity (Figure 3). Oral supplementation of L-citrulline can alleviate jejunal injury and oxidative stress in rats [87,88].

Studies have revealed that the intestinal tracts of pigs in the L-citrulline supplementation group exhibit higher microbial diversity [89]. A higher microbial diversity typically implies a more stable microbiota, and subsequently a stronger intestinal immune function. The supplementation of L-citrulline enhanced the beneficial microbiota in the intestines of iron-overloaded mice [88]. A recent investigation discovered that different feeding modalities (exclusive breastfeeding versus exclusive formula feeding) resulted in alterations in the content of citrulline and the microbiota in infant fecal samples [90]. Currently, there are relatively few studies on the influence of L-citrulline on the intestinal microbiota. In the future, it is essential to actively explore the impact of L-citrulline on the intestinal microbiota and its connection with intestinal mucosal immune functions.

Furthermore, parasitic infections can damage the intestinal mucosa and result in intestinal injury. A study on parasitic infections revealed that L-citrulline can lower the levels of IL-6 and arginase in rat serum and increase the production of NO, thereby exerting a therapeutic effect on Giardia lamblia infection [91]. The supplementation of L-citrulline can enhance the levels of interferon-γ, interleukin-10, and nitric oxide in the serum of mice infected with Toxoplasma gondii, thereby effectively treating toxoplasmosis [92]. In studies on Plasmodium, it was discovered that L-citrulline can reduce Treg cells, thereby causing an increase in the immune response and inhibiting the growth of parasites [93]. Its effects on parasitic infections (e.g., Giardia, Toxoplasma) suggest therapeutic potential, though mechanisms require further study [91,92]. It should be noted that these are animal models; human applications remain untested. Future studies should pay more attention to the therapeutic efficacy of L-citrulline on parasitic infections, particularly gastrointestinal parasites. Recent research has found that L-citrulline can inhibit ferroptosis and induce the activation of the AMPK pathway, improve the intestinal antioxidant capacity, and alleviate intestinal injury caused by iron overload [88] (Figure 3). Recent studies have also shown that L-citrulline can serve as an innate immune signaling metabolite. When pro-inflammatory stimuli occur, the consumption of L-citrulline leads to the activation of pro-inflammatory signals in macrophages [94]. Currently, the specific mechanism by which L-citrulline regulates intestinal immunity remains unclear and requires further investigation for clarification.

### 4.3. The Role of L-Citrulline in Regulating Muscle Protein Synthesis

People have constantly been exploring supplements capable of facilitating muscle protein synthesis [95], owing to the prevention of muscle atrophy caused by aging and the pursuit of physical fitness. Muscle atrophy in the elderly can severely impact the quality of life and the happiness index. Ever since Osowska et al. [96] first observed that L-citrulline could enhance nitrogen balance, L-citrulline has gradually been explored as a stimulator for protein synthesis. L-citrulline can effectively prevent skeletal muscle injury [13]. For the elderly, the supplementation of L-citrulline can ameliorate muscle protein synthesis [97]. A continuous supplementation of L-citrulline for three weeks (10 g/d) can increase the lean mass of elderly women [98]. L-citrulline can promote muscle protein synthesis in subjects with a low-protein diet [99]. Studies in vitro cell experiments have discovered that L-citrulline can increase the rate of protein synthesis in C2C12 muscle cells treated with glucocorticoids and tumor necrosis factor-α [100]. However, it is worth noting that the efficacy of L-citrulline in promoting MPS is not as good as that of branched chain amino acids (BCAAs), and its effect is not significant compared to leucine [101,102] (Table 1).

**Figure 4:**
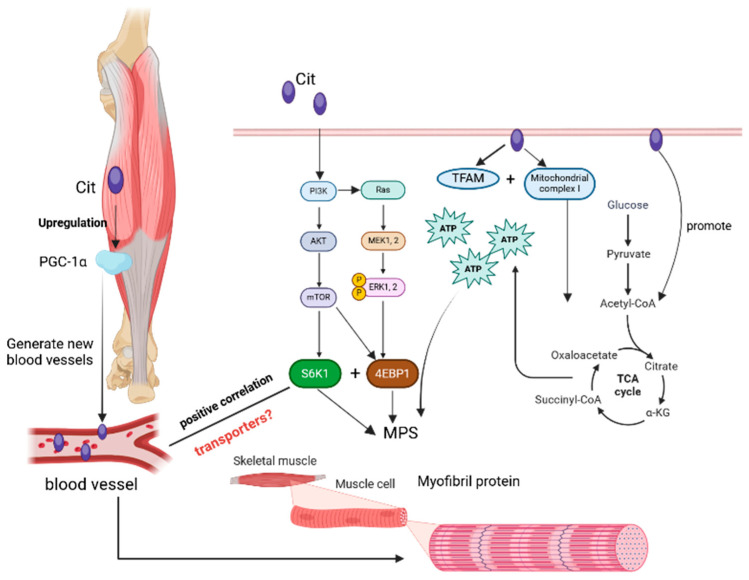
Mechanism of L-citrulline promoting muscle protein synthesis. L-citrulline regulates muscle protein synthesis by enhancing mTOR and 4EBP1 phosphorylation, acting via PI3K/MAPK/4E-BP1, correlating plasma level with S6K1 phosphorylation, influencing energy metabolism, facilitating ATP redistribution, upregulating PGC-1α for neovascularization, and acting directly not via arginine.

Extensive exploration has been conducted on the mechanism through which L-citrulline promotes muscle protein synthesis. L-citrulline enhances the phosphorylation of mTOR and 4EBP1 in mouse myocytes. However, mTORC1 inhibitors do not counteract L-citrulline-induced stimulation of muscle protein synthesis [105]. Research has indicated that the regulatory action of L-citrulline on the protein anabolic metabolism of muscle cells is independent of the insulin and Akt pathways, and it directly regulates muscle protein synthesis in rats via the PI3K/MAPK/4E-BP1 pathway [101] (Figure 4). Additionally, the authors observed that the L-citrulline concentration within rat muscles has no association with the phosphorylation of muscle S6K1, but the L-citrulline concentration in plasma shows a positive correlation with the phosphorylation of muscle S6K1. Based on this consideration, it is necessary to contemplate what leads to the variations in L-citrulline content in plasma and muscles. Hence, in the future, the role of L-citrulline transporters in muscles should be explored. L-citrulline is closely intertwined with energy metabolism. In proteomic studies on rats, L-citrulline can stimulate the expression of mitochondrial biogenesis factor (TFAM: mitochondrial transcription factor A) and the activity of mitochondrial complex I [106]. Another study demonstrated that mitochondrial metabolism is not directly modulated by L-citrulline supplements. Nevertheless, L-citrulline supplements can activate the pathway generating acetyl-CoA, thereby influencing energy metabolism [107]. Recent research has revealed that the mechanism by which L-citrulline promotes muscle protein synthesis might be its facilitation of ATP redistribution to the protein synthesis process [102]. Considering that the protein synthesis process demands a considerable amount of ATP, in the future, the focus should be on exploring the regulation of L-citrulline on mitochondrial function and the connection between this regulation and protein synthesis, as this is intimately related to the combined efficacy of L-citrulline and energy supplements. An increase in blood flow within muscles can boost protein synthesis, and L-citrulline can upregulate the expression of peroxisome proliferator-activated receptor-γ coactivator-1α (PGC-1α), which is regarded as associated with neovascularization [108]. However, while a pilot human study showed increased MPS with 3–6 g/day in low-protein diets [99], subsequent human trials are limited and inconsistent; arginine supplementation or blood flow modulation has not consistently improved MPS, suggesting mechanisms may not involve NO alone [102]. Claims should be viewed as promising but requiring more robust human evidence. Studies have found that L-citrulline promotes muscle protein synthesis directly rather than being metabolized into arginine first [100], thus the conventional perception of L-citrulline (that L-citrulline promotes protein synthesis in relation to arginine) might be overturned.

## 5. The Beneficial Effects of L-Theanine

Building on citrulline’s effects, this section parallels L-theanine’s roles in immunity, anti-tumor activity, and antioxidants, highlighting shared immune and oxidative stress themes for a cohesive narrative.

### 5.1. The Role of L-Theanine in Regulating Intestinal Immunity

**Figure 5:**
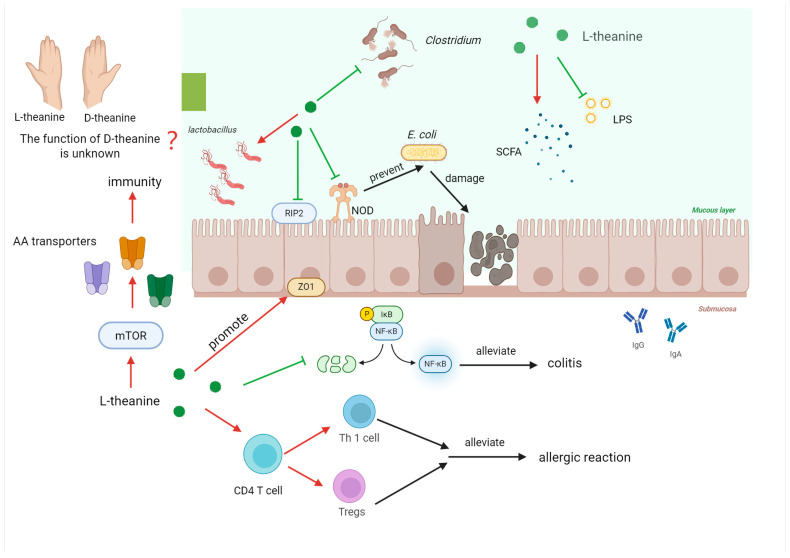
Mechanism of L-Theanine enhancing intestinal immunity. L-theanine enhances intestinal immunity via two main mechanisms. It regulates signaling pathways, inhibiting NOD—like receptors, RIP2, NF-κB, and p38 MAPK/NLRP3. It promotes CD4^+^ T cell differentiation, upregulates tight—junction proteins, and increases amino—acid transporters via mTOR. It also modulates the intestinal microbiota, promoting beneficial bacteria growth, increasing short-chain fatty acids, and acting in an MHC-II-dependent manner. Research on D-theanine’s immune—regulatory function and theanine chiral—structure effects remains scarce.

Long-term tea consumption confers numerous benefits on the human body, exerting improving effects on antioxidation, anti-inflammation, immune capacity, cardiovascular diseases, diabetes, and obesity, among others [109]. L-theanine, as the predominant free amino acid in tea, has naturally attracted people’s attention. L-theanine can regulate intestinal immune capacity via multiple signaling pathways (Figure 5). It can decelerate intestinal infection of mice caused by Escherichia coli by inhibiting the expression of nucleotide-binding oligomerization domain (NOD) like receptors and RIP2 [110]. Another study indicates that L-theanine can facilitate the differentiation of CD4^+^ T cells in the jejunum of mice into Th1 and Tregs, thereby mitigating intestinal allergic reactions in mice [111]. Research conducted on piglets has revealed that L-theanine can increase the expression of tight junction proteins to alleviate intestinal inflammatory responses in piglets [112]. L-theanine can alleviate colonic inflammation in mice by down-regulating the NF-κB signaling pathway and regulating lipid metabolism disorders [113]. Furthermore, studies have disclosed that L-theanine can inhibit the expression of nitric oxide synthase, cyclooxygenase-2, TLR-2, TLR-4, TLR-6, and TLR-9 in mice, increase short-chain fatty acids, and reduce the production of lipopolysaccharides to ameliorate colonic inflammation [114]. In a model of LPS-induced intestinal mucosal damage in piglets, it was found that L-theanine could down-regulate the p38 MAPK/NLRP3 signaling pathway to reduce the impairment of tight junction proteins, thereby enhancing the intestinal immune function of piglets [115]. In rats induced by a high-fat diet, it was discovered that L-theanine can alleviate colitis through the MAPK/NF-κB signaling pathway [116]. Recent research has uncovered that L-theanine can increase the expression of amino acid transporters in the small intestine of mice via the mTOR signaling pathway, and the content of amino acids in the intestine is closely associated with immune capacity [117].

The regulation of immunity by intestinal microbiota has always been a hot research field. L-theanine can regulate intestinal immunity by influencing the composition of intestinal microbiota. Studies on broilers have found that L-theanine can promote the proliferation of lactic acid bacteria in the intestine and reduce the proliferation of harmful bacteria such as Clostridium to enhance the intestinal health of broilers [118]. Research has disclosed that L-theanine can increase the proportions of *Prevotella*, *Lachnospira*, and *Ruminococcus* in the rat intestine and increase the content of short-chain fatty acids (acetic acid, propionic acid, and butyric acid) to promote intestinal immunity [119]. In rats induced by a high-fat diet, it was found that L-theanine can increase the abundance of *Blautia coccoides* and *Lactobacillus murinu*, which is beneficial for the alleviation of colitis [116]. A recent study has discovered that L-theanine can regulate the intestinal microbiota of mice and alleviate colitis in an MHC-II-dependent manner [120]. These effects are mostly from animal models; human studies are scarce but suggest potential via tea consumption for gut health. Currently, most studies focus on the regulatory effect of L-theanine on intestinal immunity and its mechanism. People have limited knowledge about D-theanine. Research has found that the metabolic pathways of D-theanine and L-theanine in rats are different, suggesting that the functions of the two enantiomers may vary [121]. It is unknown whether D-theanine also possesses immune regulatory functions. Future research should pay attention to the regulation of the chiral structure of theanine on the immune capacity of the organism. The role of D-theanine in immunity remains understudied, and chiral-specific effects need exploration [121].

### 5.2. The Role of L-Theanine in Inhibiting Tumors

Cancer has always been the greatest threat to human life, and the entire world is dedicated to developing drugs capable of treating cancer, particularly those naturally occurring ones [122]. Some recent in vitro and in vivo studies have indicated that L-theanine possesses certain anti-tumor effects [123,124,125] and has no detrimental impact on normal cells [126]. However, human evidence is lacking; effects are dose-dependent and not observed at low doses/short terms. However, there are certain disputes regarding the anti-tumor effects of L-theanine. For instance, no beneficial effects of L-theanine against tumor occurrence were observed in studies with low doses and short-term interventions [127,128,129], which might be attributed to the dose variations and usage duration of L-theanine. Additionally, L-theanine offers certain benefits in alleviating the side effects caused by tumor chemotherapy. For example, L-theanine can mitigate the renal toxicity induced by DOX (a widely utilized chemotherapy agent) [130]. L-theanine can alleviate the adverse events of S-1 adjuvant chemotherapy in patients with gastrointestinal cancer [130]. Oral administration of L-theanine and cysteine can reduce the incidence of diarrhea in colorectal cancer patients after receiving capecitabine adjuvant chemotherapy [131]. Hence, L-theanine can also serve as an adjunctive medication for chemotherapy in the future.

L-theanine can inhibit the proliferation of tumor cells through multiple pathways. Research has disclosed that L-theanine can prevent the occurrence of cervical cancer by inhibiting the EGFR/Met-Akt/NF-κB signaling pathway [132]. Studies on in vitro models of hepatoma cells have revealed that L-theanine can significantly suppress the Met/EGFR/VEGFR-Akt/NF-κB pathway, thereby inhibiting the growth of hepatoma cells [133]. L-theanine can inhibit the metastasis of prostate cancer by inhibiting the ERK/NF-κB signaling pathway [134]. Research has found that L-theanine and theobromine, either alone or in combination, can inhibit the Ki-67 and Akt/mTOR pathways to prevent the occurrence of colon cancer [125]. Activating and promoting the apoptosis of cancer cells is another potential approach in cancer treatment. A study on hepatoma cells has found that L-theanine can effectively induce the apoptosis of hepatoma cells through the mitochondrial pathway under conditions of glutamine deficiency [135]. Considering that the structure of L-theanine is highly similar to that of glutamate [28], and increasing the intake of glutamate can reduce the risk of colorectal cancer in non-overweight individuals [136], it remains necessary to conduct further research to determine whether the anti-tumor effect of L-theanine is related to glutamate. Although the anti-tumor effect of L-theanine has been acknowledged at present, the dosage, duration of use, and effects on different types of cancer still require further exploration. In the future, the therapeutic effects of L-theanine derivatives on tumors should also be investigated. Additionally, the anti-tumor function of D-theanine should be further studied, as there is currently very limited knowledge about it [123].

### 5.3. The Antioxidant Function of L-Theanine

**Figure 6:**
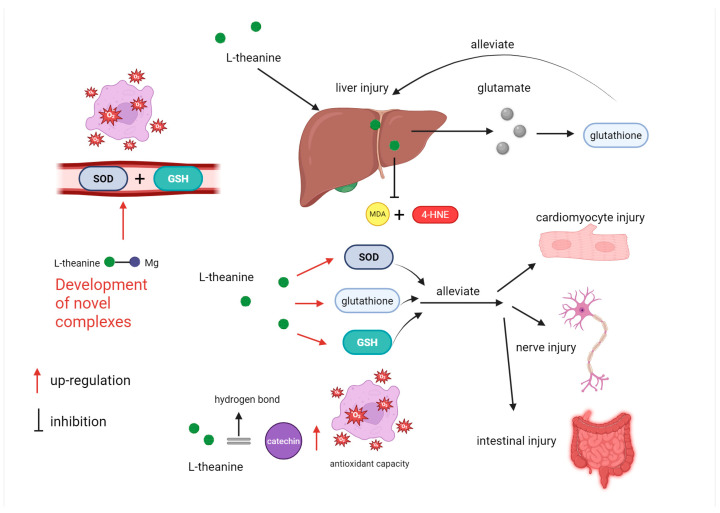
The antioxidant function of L-theanine. L-theanine, a bioactive component in tea, exhibits potent antioxidant properties. In the liver, it is metabolized to glutamic acid, contributing to glutathione synthesis. It alleviates oxidative-stress-induced injuries in multiple organs. In the liver, it regulates Nrf2 signaling, inhibits lipid peroxidation, and boosts glutathione levels to counteract EGCG-induced injury and E. coli-infection-related oxidative stress. In myocardial cells, it enhances antioxidant enzyme activities to relieve hydrogen-peroxide—and ischemia–reperfusion-induced damage. In the nervous and intestinal systems, it reduces oxidative stress markers and improves antioxidant capacity. Moreover, L-theanine can enhance the antioxidant capacity of catechins via hydrogen bonding and of diglycerides through hydrophobic binding. Novel complexes like Mg-L-theanine increase serum antioxidant enzyme activities. Future research should focus on its synergistic effects with other antioxidants and development of new complexes.

Tea has long been regarded as an excellent antioxidant, which is attributed to its bioactive substances such as L-theanine, alkaloids, and tea polyphenols [137]. L-theanine is metabolized in the liver to generate glutamic acid, which can participate in the synthesis of glutathione, an important antioxidant [138]. L-theanine can alleviate various diseases resulting from oxidative stress, such as liver injury [139,140], neuronal damage [34,141], and myocardial damage [142,143], through its antioxidant capacity (Figure 6). A study asserts that L-theanine can enhance the antioxidant activity of the liver in mice infected with *Escherichia coli* [31]. After epigallocatechin-3-gallate (EGCG) induces liver injury in mice, lipid peroxidation products such as MDA and 4-HNE accumulate in the liver. L-theanine inhibits the excessive generation of these lipid peroxidation products and alleviates liver injury in mice by regulating Nrf2 signaling and increasing glutathione content [144]. L-theanine enhances glutathione levels as well as the activities of glutathione peroxidase and superoxide dismutase, thereby alleviating hydrogen peroxide-induced myocardial cell damage by improving antioxidant capacity [145]. Reperfusion after myocardial ischemia can alleviate myocardial injury, but reperfusion increases the production of reactive oxygen species. A study has discovered that L-theanine can increase the activity of antioxidant enzymes and effectively mitigate oxidative stress [143]. L-theanine can improve the pathological changes caused by nerve injury by reducing the oxidative stress levels (NO, MDA) in the sciatic nerve of rats and increasing antioxidant capacity (GSH, SOD, CAT) [146]. Oxidative stress causes intestinal injury in weaned piglets, and the use of antioxidants can alleviate weaning stress to a certain extent. L-theanine can increase the activity of antioxidant enzymes in piglets, reduce the MDA content in jejunal mucosa, and alleviate hydrogen peroxide-induced intestinal cell damage in piglets by activating the Nrf2 signaling pathway and reducing keap1 expression [142,147].

Apart from its own antioxidant ability, the latest research has discovered that L-theanine can enhance the antioxidant capacity of catechins through the hydrogen bonding force between L-theanine and catechin molecules [148]. L-theanine can increase the antioxidant capacity of diglycerides by hydrophobic binding with lactoglobulin [149]. In the future, attention should also be given to the synergistic effect of L-theanine and other antioxidants as well as their application in disease treatment. The novel complex of theanine: Mg-L-theanine has been proven to increase the activity of antioxidant enzymes in serum [150]. The development of new complexes of L-theanine in the future will also be an effective approach to fully exploit its antioxidant capacity.

## 6. Limitations, Potential Side Effects, and Considerations for Use

While L-citrulline and L-theanine show promise, limitations include sourcing challenges (e.g., citrulline from watermelon rinds, variable yields 0.7–3.6 g/kg; theanine from tea leaves, 1–3% dry weight), availability as supplements (quality varies by manufacturer), and potential side effects. Citrulline is generally safe up to 15 g/day in humans, but high doses (>10 g) may cause gastrointestinal upset (e.g., bloating, diarrhea) or hypotension; long-term use lacks data, and it may interact with blood pressure medications [68]. Theanine is safe up to 900 mg/day for 8 weeks, with rare mild effects like headache, nausea, or irritability; high doses may lower blood pressure or interact with stimulants [126]. Benefits in physiological settings are best supported in humans for cardiovascular/NO effects (citrulline) and stress reduction (theanine), but proof requires more RCTs. Increasing via diet (e.g., watermelon for citrulline, tea for theanine) may not achieve therapeutic levels without supplements; deficits link to disease but supplementation benefits vary by individual. Overall, while efficacious in animals/cells, human applications need caution regarding dose (3–6 g citrulline, 200–400 mg theanine) and long-term safety.

## 7. Conclusions

**Figure 7:**
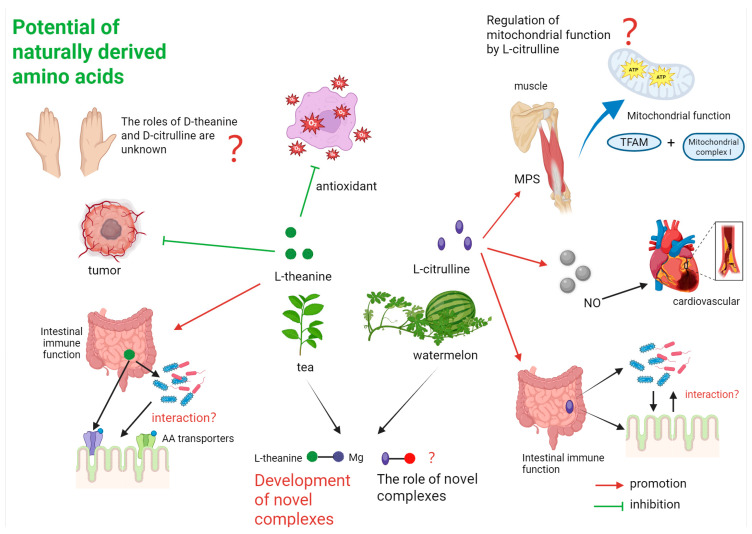
Possible future research hotspots of L-citrulline and L-theanine.

L-citrulline and L-theanine exhibit promising regulatory effects on human health, particularly in cardiovascular, immune, and antioxidant functions. However, their benefits, such as L-citrulline’s role in muscle protein synthesis, are less pronounced than those of established nutrients like leucine, and claims of anti-tumor effects for either compound lack clinical validation, and require further research. Future research should explore D-amino acid metabolism, chiral-specific effects, and the development of derivatives and complexes. The interplay between L-citrulline’s NO production and arginine’s polyamine synthesis, as well as L-theanine’s mitochondrial and immune modulation, warrants further investigation to optimize their nutraceutical applications (Figure 7).

## Figures and Tables

**Table 1:** Comparison of L-Citrulline and Leucine in Muscle Protein Synthesis.

Amino Acid	Mechanism	Efficacy	Key Studies
L-Citrulline	Activates PI3K/MAPK/4E-BP1, enhances ATP redistribution, upregulates PGC-1α	Modest, less potent than BCAAs	[98,101,102]
Leucine	Strongly activates mTOR, promotes protein synthesis	Highly effective, gold standard	[103,104]
